# Errata

**Published:** 1859-07-01

**Authors:** 


Errata: No. XIV.—Expunge the inverted commas at the commencement and termination of the first and second paragraphs on page 283; the paragraphs in question not being quotations from Mr. Fletcher's work, but an abstract of certain portions of it. At p. 292, l. 17, for Forgier read *Fregier*; and add to the *causes* of insanity in Ireland, p. 309, *religious feelings*, 45 males and 59 females.

